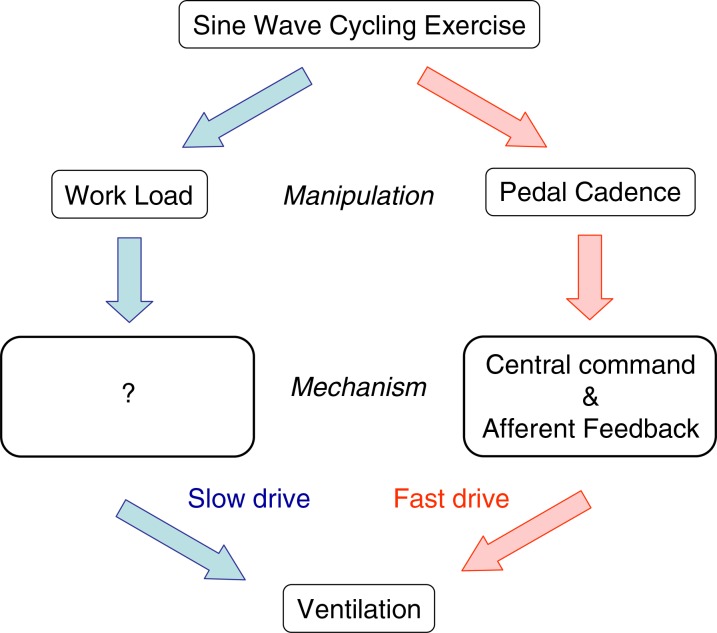# The fast exercise drive to breathe

**DOI:** 10.1113/jphysiol.2013.258897

**Published:** 2013-09-11

**Authors:** James Duffin

**Affiliations:** Departments of Anaesthesia and PhysiologyUniversity of Toronto

## Abstract

This paper presents a personal view of research into the exercise drive to breathe that can be observed to act immediately to increase breathing at the start of rhythmic exercise. It is based on a talk given at the Experimental Biology 2013 meeting in a session entitled ‘Recent advances in understanding mechanisms regulating breathing during exercise’. This drive to breathe has its origin in a combination of central command, whereby voluntary motor commands to the exercising muscles produce a concurrent respiratory drive, and afferent feedback, whereby afferent information from the exercising muscles affects breathing. The drive at the start and end of rhythmic exercise is proportional to limb movement frequency, and its magnitude decays as exercise continues so that the immediate decrease of ventilation at the end of exercise is about 60% of the immediate increase at the start. With such evidence for the effect of this fast drive to breathe at the start and end of rhythmic exercise, its existence during exercise is hypothesised. Experiments to test this hypothesis have, however, provided debatable evidence. A fast drive to breathe during both ramp and sine wave changes in treadmill exercise speed and grade appears to be present in some individuals, but is not as evident in the general population. Recent sine-wave cycling experiments show that when cadence is varied sinusoidally the ventilation response lags by about 10 s, whereas when pedal loading is varied ventilation lags by about 30 s. It therefore appears that limb movement frequency is effective in influencing ventilation during exercise as well as at the start and end of exercise.

## Introduction

Ventilation responds immediately at the onset of exercise on both a cycle ergometer (Krogh & Lindhard, 1992) and a treadmill (Duffin & Bechbache, 1959). In the late 1950s, Dejours *et al*. (1964) studied the breathing response to steady-state rhythmic exercise and concluded that two drives to breathe existed. The fast and slow components of the ventilatory response were hypothesised to result from neural and chemical drives to breathe respectively (Dejours, 1987). It is Dejours’ neural drive or the more recently termed phase 1 of exercise hyperpnoea (Whipp, 1981) that I term the fast exercise drive of the title.

**Figure 1:**
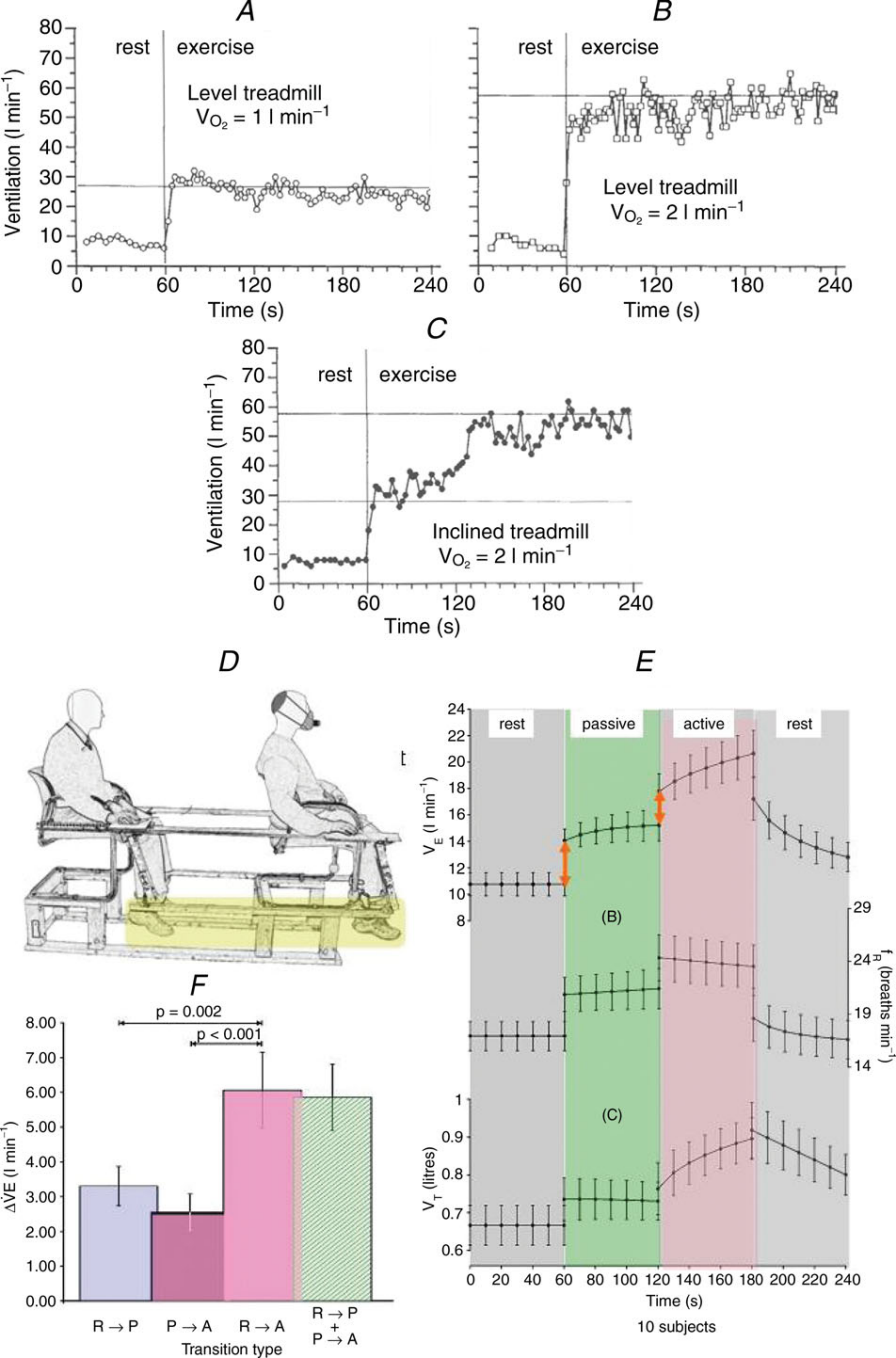
*A*, level treadmill at a slow speed requiring a 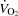 = 1 l min^−1^. *B*, level treadmill at a faster speed requiring a 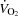 = 2 l min^−1^. *C*, inclined treadmill at the same speed as in *A* requiring a 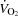 of 2 l min^−1^. *A–C* are excerpts from Casey *et al*. (1977). *D*, the tandem chair couples the legs of the experimenter and subject (yellow highlight) and allows the experimenter seated behind the subject to move the subject's legs passively. *E*, 10 s binned mean (SEM) results from 10 subjects showing the transitions: ventilation (top), breathing frequency (middle) and tidal volume (bottom). At both transitions (red arrows) first from rest to passive movement and then from passive movement to active exercise, ventilation increases quickly. *F*, the 10-subject mean (SEM) changes in breathing at the exercise transitions shows that rest to passive movement (R-P), and passive to active movement (P-A) sum (R-P + P-A) to equal the rest to active exercise transition (R-A). *E* and *F* are excerpts from Bell & Duffin (2006).

Further investigations in the 1980s in my laboratory confirmed the findings of other investigators (e.g. Dejours *et al*. 1964; D'Angelo & Torelli, 1978) that the fast drive to breathe at the start of exercise is proportional to limb movement frequency: cadence on a cycle ergometer and pace on a treadmill. Figure 1*A*, *B* and *C* shows one such experiment (Casey *et al*. 1977) conducted on a treadmill with an immediate start capability.

As a comparison of Fig. 1*A* and *B* shows, when the treadmill speed is increased the sudden increase in ventilation at the start of walking also increases. Keeping the slow walking pace of Fig. 1*A* but inclining the treadmill so that the load is increased to that of Fig. 1*B* is shown in Fig. 1*C*. The immediate increase in ventilation at the start of exercise is the same as that observed in Fig. 1*A*. However, the initial increase in ventilation at the start of exercise does not provide sufficient gas exchange for this workload and so ventilation increases slowly to reach the same level as the previous 2 l min^-1^ exercise on the level treadmill at the faster speed shown in Fig. 1*B*. Figure 1*C* therefore illustrates the origin of the fast and slow exercise drives nomenclature. Note that the name fast exercise drive does not imply that the origin is neural or that the drive is confined to phase 1 of the hyperpnoea. These experiments therefore showed that treadmill speed influenced the magnitude of the increase in ventilation at the start of exercise, confirming the influence of limb movement frequency but not load on the fast exercise drive. They also demonstrate that there is a slower drive relating to work intensity.

## Origin of the fast exercise drive to breathe

The origin of the fast exercise drive to breathe has been debated since the beginning of exercise physiological investigations more than a century ago, where two theories were proposed. The fast exercise drive was thought to originate from a parallel stimulation of respiratory neurons in the medulla by limb movement motor commands via the hypothalamus, a hypothesis known as central command. Alternatively, stimulation of respiratory neurons in the medulla was by group III and IV afferents from the moving limbs, a hypothesis known as peripheral afferent feedback. There seemed to be no way of determining which of these hypotheses was correct and a number of experimental paradigms were tried (see the review by Mateika & Duffin, 1913). One promising line of experimentation involved passive exercise, where the limbs are moved passively so that only afferent feedback occurs. Using a tandem chair apparatus that allows the investigator to passively move a subject's legs or vice versa Bell & Duffin (2006) showed that both hypotheses were correct and contributed to the fast exercise drive at the start of rhythmic exercise (Fig. 1*D* and *E*).

Using the tandem chair and passively moving the subject's legs produces an immediate increase in ventilation, and ventilation also increases abruptly when the subject assumes voluntary control of the leg movements. The bar graph in Fig. 1*F* shows that the sum of these rest-to-passive and passive-to-active abrupt changes in ventilation equals the change in ventilation as the subject starts the exercise from rest. Thus, it appears that both central command and afferent feedback play a role in determining the fast exercise drive to breathe.

## Characteristics of the fast exercise drive to breathe

**Figure 2:**
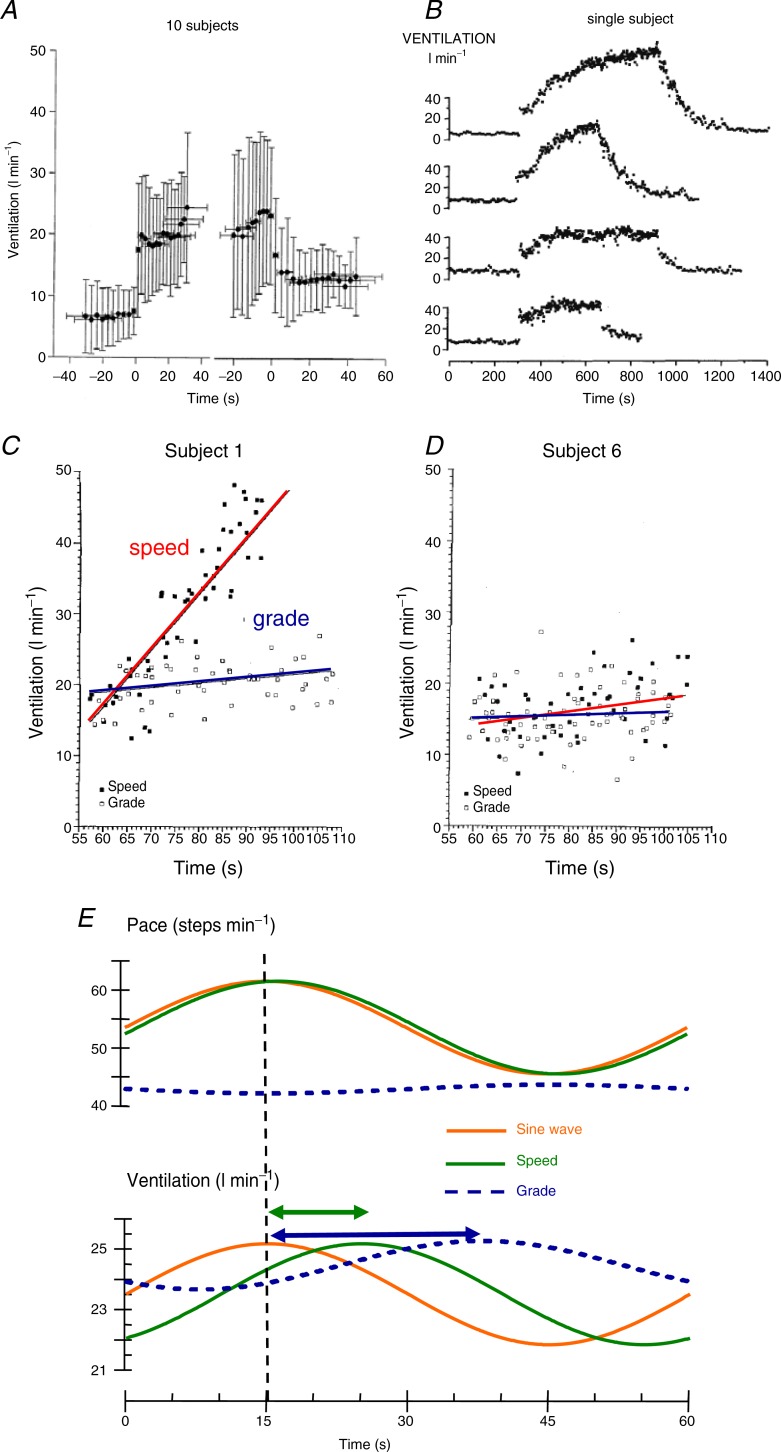
*A*, breath-by-breath changes in ventilation at the start and end of moderate exercise of 1 min duration; ensemble averages and standard deviations for 10 subjects (reproduced from Koehle & Duffin (1983) with permission). *B*, breath-by-breath ventilation for a single subject at four levels of exercise. The levels of exercise expressed as percentage of 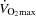 from top to bottom are: 80%, 10 min; 80%, 6 min; 50%, 10 min; 50%, 6 min (reproduced from Mateika & Duffin (1996) with permission). *C* and *D*, ventilation changes for two subjects during ramp changes in exercise load produced by increasing treadmill speed (filled squares and red lines) or by increasing treadmill grade (open squares and blue lines) to equivalent end-points of oxygen uptake. The points are breath-by-breath values and the lines are fitted by linear regression (reproduced from Kelsey & Duffin (1963) with permission). *E*, average (19 subjects) fitted sine waves for pace and ventilation during sine wave treadmill exercise (extract from Wells *et al*. 1992). The treadmill sine wave variation of either speed or grade is shown as a red line. The responses are shown as green for treadmill speed and blue for treadmill grade.

A series of experiments in my laboratory explored the characteristics of the fast exercise drive to breathe (Mateika & Duffin, 1913). As noted by others before us (e.g. Dejours, 1971) the fast increase in ventilation at the start of exercise is mirrored by a fast decrease in ventilation as exercise ceases. However, the decrease in ventilation at the end of exercise is less than that at the start, and the ventilation fall at the end of exercise is only about 60% of the rise at the start, even if exercise is of short duration such as 1 min, as shown in Fig. 2*A*. Thus, the fast exercise drive does not remain constant throughout exercise but decays quickly (within 1 min; Koehle & Duffin, 1983).

These findings for moderate exercise also apply for longer moderate exercise bouts (Mateika & Duffin, 1996) as shown by the lower two charts in Fig. 2*B*. However, during heavy exercise, the fast increase in ventilation at the start of exercise has virtually disappeared by the end, as shown in the top two charts in Fig. 2*B*. It therefore would appear that the fast exercise drive to breathe is present during moderate exercise, as it appears at the start of exercise and disappears at the end, but it decays rapidly and disappears during heavy exercise.

## Detecting the presence of the fast exercise drive to breathe during exercise

As the ventilation changes abruptly at both the start and the end of moderate rhythmic exercise it appears that the drive must be maintained throughout exercise; but how could its presence be detected during exercise? If ventilation transitions are proportional to changes in limb movement frequency, then we hypothesised that changing the frequency of limb movement during exercise would affect ventilation. These experiments involved changing the exercise workload either by altering limb loading (grade on a treadmill or pedal load on a cycle ergometer), or by adjusting the rhythm of exercise (speed/pace on a treadmill or pedalling speed/cadence on a cycle ergomenter).

Ramp changes between two load levels achieved either by adjusting treadmill speed or grade suggested that the fast exercise drive was active throughout exercise as hypothesised, but was more apparent in some subjects than others (Kelsey & Duffin, 1963). In these experiments subjects walked on a treadmill for two tests. In one test the exercise load was increased by increasing the speed and in the other test the grade was slowly increased. The start and end speeds and grades were adjusted so that the oxygen consumptions at the start and end were the same. Figure 2*C* and *D* illustrates results from these tests, with one subject confirming the hypothesis that an increasing pace increased ventilation and another not. A better way of assessing the presence of the fast exercise drive to breathe was needed.

Experiments employing a sine-wave adjustment of cycle ergometer exercise load were pioneered by Casaburi *et al*. (2006). Their experiments varied the workload by pedal loading at constant cadence, and with a 2 min sinusoidal period the ventilation changes lagged behind the load changes by about 40 s. A year later they repeated these experiments, but varied the cadence (Casaburi *et al*. 2006). This time the ventilation changes during a sinsusoidal variation of cadence with a 2 min period followed more closely, lagging by about 13 s. These results were interpreted by us (but not by them) as supporting the continuing influence of the fast exercise drive during exercise. Some 30 years later the same type of comparison but using a treadmill found much the same result (Wells *et al*. 1992). Figure 2*E* shows that the grade response has a lower amplitude and greater lag than the speed response.

**Figure 3:**
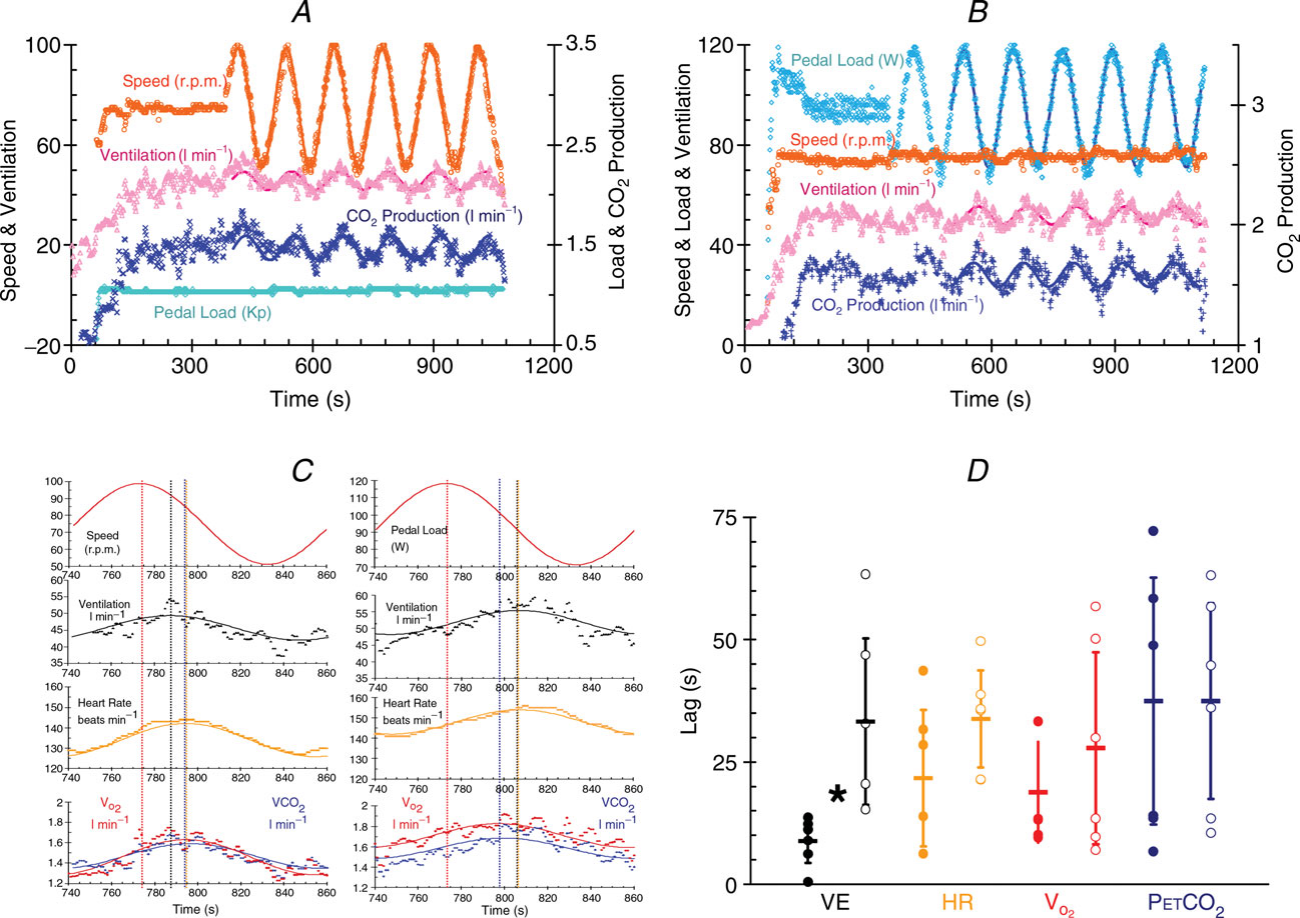
*A*, breath-by-breath variables measured during an exercise test with the subject varying pedalling speed (cadence) while pedal loading remains constant (extract from a 2013 poster by Caterini *et al*. in *Experimental Biology*). The solid lines are fitted sine waves. *B*, breath-by-breath variables measured during an exercise test with varying pedal loading while pedalling speed (cadence) remains constant (extract as above). The solid lines are fitted sine waves. *C*, a single sine wave taken from the tests shown in *A* and *B* (extract as above). On the left, speed or cadence is changed sinusoidally while pedal loading is constant (top red trace), and on the right pedal loading is changed sinusoidally while cadence is constant (top red trace). The traces below show breath-by-breath ventilation (black symbols), heart rate (orange symbols), oxygen uptake (red symbols) and carbon dioxide production (blue symbols). The solid lines are the fitted sine waves and the vertical lines show the timing of the peaks of the fitted responses. *D*, sinusoidal exercise time lags. The time lag for ventilation (black symbols), heart rate (orange symbols) and oxygen consumption (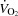, red symbols) behind the sine wave variations in cadence at constant pedal loading (filled symbols) and the sine wave variations in pedal loading at constant cadence (open symbols) measured during the sine wave tests (extract as above). The means are indicated as horizontal bars and the standard deviations as vertical lines. *Significant difference.

Modern cycle ergometers are capable of maintaining a constant pedal load during cadence changes as well as being able to alter pedal loading under computer control. Jessica Caterini, Greg Wells and I therefore began experiments to re-examine sine wave alterations in cadence and the effect on ventilation, testing for the appearance of the fast drive to breathe during cycle ergometer exercise. Subjects alter their cadence sinusoidally with a 2 min period while pedal loading remains constant. Oxygen consumption does vary, and so for comparison, subjects undergo a second test in which pedal loading is changed sinusoidally while cadence remains constant. The oxygen consumption changes are adjusted to be similar for each test. Examples of the two testing regimes are shown in Fig. 3*A* and *B*. Note that the protocol has a warm up period before sinusoidal changes begin that is sufficient to allow the reduction of the fast drive to about 60% of its initial value (Fig. 2*A*).

Figure 3*C* shows close-up views of a single sine wave for these same experiments. On the left, speed or cadence is changed sinusoidally (top red trace) and on the right pedal loading is changed sinusoidally (top red trace). The traces below show ventilation, heart rate, oxygen uptake and carbon dioxide production on a breath-by-breath basis with fitted sine waves. Vertical lines are drawn through the peaks to show their alignment. When cadence changes sinusoidally, ventilation changes precede heart rate and metabolism changes, which are closely aligned. When pedal loading changes sinusoidally, metabolism changes precede ventilation and heart rate, which are closely aligned. These experiments are in progress and so far only six subjects have participated. Figure 3*D* shows that the lag of ventilation behind the sine wave changes is much less for the cadence variation than for the pedal load variation and is about 10 s compared to 30 s.

## Conclusion

The current preliminary results are very similar to those obtained using the treadmill previously and the early cycle ergometer experiments. We provisionally conclude that the fast drive to breathe is present during exercise and responds to the rhythm of limb movements and Figure 4 shows a summary of our current hypotheses. We also note that the cadence variation reduces the lags of heart rate and oxygen consumption, and that end-tidal CO_2_ tension lags appear unrelated to ventilation lags. Finding the mechanisms underlying these observations will require some ingenious experimental manipulations, but it may be possible to partition the central command and afferent feedback components, and discover the influence of the speeded up circulation. Such sine wave manipulations of exercise parameters may offer new insights into the hyperpnoea of exercise.

**Figure 4:**